# Proving of Apiol (Oil of Parsley)

**Published:** 1885-10

**Authors:** Samuel Swan

**Affiliations:** New York


### PROVING OF APIOL (OIL OF PARSLEY).

SAMUEL SWAN, M. D., NEW YORK.

Mrs. M. B. P. took *Apiol* $^{50m}$ in January, 1880; the following symptoms were elicited: [NOTE.—In this and other of my provings, symptoms purely clinical are marked °; pathogenitic symptoms, clinically verified, are marked *.]

°Nothing worries her, is better-natured than usual.

Frequently dizzy.

When reading, the right page laps over on the left; but not *vice versa.*

Appetite excellent.

Inclined to nausea in afternoon; is not sick at stomach, but feels as if she would be.

Inclined to constipation.

Urinates every five minutes, not much at a time; constant inclination; color bright lemon.

Menses slight, no pain, was not aware of their occurrence.

Sudden palpitation of heart when quiet or at night, as if she had been frightened or had run up-stairs, followed by frequent sighing; after it passes off is very hot, face flushed, and head seems swelled; then white mucus from vagina, drying hard.

When walking, pain across hollow of left foot; worse when standing, when it feels like a stitch.

Restless, cannot sleep before one or two A. M., or if she falls asleep before midnight she wakes at that time, and then cannot sleep till daylight.

Can only get to sleep by keeping the hand or foot in motion.

